# Comprehensive cDNA cloning and putative feature analysis of endogenous cellulases possessed by the Pacific oyster, *Crassostrea gigas*

**DOI:** 10.1371/journal.pone.0313246

**Published:** 2025-02-07

**Authors:** Manabu Wen-Liu Tanimura, Kazuhiko Koike, Motoki Kayama, Kazumi Matsuoka

**Affiliations:** 1 Graduate School of Human and Environmental Studies, Kyoto University, Sakyo, Kyoto, Japan; 2 Seed Bank Co., Ltd. Sakyo, Kyoto, Japan; 3 Graduate School of Biosphere Science, Hiroshima University, Higashi-Hiroshima, Hiroshima, Japan; 4 C/O Institute for East China Sea Research, Nagasaki University, Nagasaki, Japan; Newcastle University, UNITED KINGDOM OF GREAT BRITAIN AND NORTHERN IRELAND

## Abstract

Previous studies have examined the cellulase activity of *Crassostrea gigas* (Pacific oyster) and suggested its potential utilization of terrestrial lignocellulose. However, no studies have been conducted to comprehensively assess its endogenous cellulases. Therefore, our objective was to identify the cellulases present in *C*. *gigas* through transcriptome and genomic analyses. The results showed that there are 10 cellulase orthologs, seven of which are endogenous. Phylogenetic analysis revealed that two of these cellulases belong to the glycoside hydrolase family (GHF) 5, four to GHF9, and one to GHF45. An alignment of the amino acid sequences suggested the presence of at least endo-β-1,4-glucanase. Therefore, *C*. *gigas* is likely capable of decomposing lignocellulose into glucose. This finding supports the fact that *C*. *gigas*, a globally commercial bivalve species, thrives in environments that lack phytoplankton, such as mangroves.

## Introduction

The Pacific oyster, *Crassostrea* (*Magallana*) *gigas* (Thunberg, 1793), originated from East Asia but is now becoming the most widely cultured commercial species worldwide [1]. It feeds on various types of food, including benthic and planktonic diatoms and dinoflagellates [2]. A diet consisting of phytobenthos and phytoplankton is rich in lipids, which are considered essential for the survival of their larval stage and the growth of the adult stage. In contrast, Japanese fishermen have observed that upstream forestation could increase the productivity of oysters cultured downstream. Initially, this was explained as a result of the inflow of inorganic particles, such as iron and phosphates, from upstream forests, which increases the production of phytoplankton. In the mangrove areas of Southeast Asia, large amounts of sediments are supplied from the land during the rainy season, resulting in high turbidity that hinders phytoplankton growth in these estuarine areas. Therefore, it is highly likely that heterotrophic organisms, including oysters inhabiting mangrove areas during the rainy season, feed on substances other than phytoplankton [3]. This suggests that oysters could utilize terrestrial organic matter as an alternative nutrient source.

Cellulases play a crucial role in hydrolyzing beta-glucoside linkages in lignocellulose, which is the primary component of plant cell walls and the most abundant organic material on Earth. Initially, microorganisms such as bacteria, fungi, and protozoa were studied as species capable of feeding on lignocellulose [4]. It was also believed that herbivorous invertebrates symbiotically associated with these microorganisms, particularly bacteria that possessed cellulases, to aid in cellulose digestion [5]. However, Watanabe et al. later discovered that the Japanese termite, *Reticulitermes speratus*, actually carries a cellulase gene on its chromosome [6]. Since then, various invertebrates, including bivalves and gastropods, have been found to possess endogenous cellulases [7]. Stable isotope analyses have shown that oysters can assimilate terrestrial cellulose [8]. This suggests that oysters might also use terrestrial cellulose as a food source. Given the growing significance of *C*. *gigas* in aquaculture economics, it is crucial to thoroughly understand its feeding habitat in order to improve production.

To date, only a few studies have investigated the endogenous cellulases of oysters, with the exception of the mangrove oyster, *C*. *rivularis* [9]. Moreover, despite the importance of revealing endogenous cellulases for a better understanding of the physiology and ecology of aquatic invertebrates, few studies have provided an overview of specific endogenous cellulases in invertebrates, except for the Japanese local clam, *Corbicula japonica*. Previous studies have identified multiple endogenous cellulases by searching for their cDNA using degenerate primers designed based on amino acid sequences determined through protein separation and zymographic analyses [10–12]. However, this cascade of procedures is neither sufficient nor completely reliable because cellulases with similar molecular weights cannot be fully separated. In the present study, we conducted a comprehensive investigation of the endogenous cellulases of *C*. *gigas* by evaluating total mRNA and using both cDNA and genomic DNA of *C*. *gigas* to verify the transcriptome results. Additionally, we utilized the Carbohydrate-Active Enzyme (CAZy) database to collect and construct a eukaryotic endogenous cellulase library in order to perform phylogenetic analysis of the detected cellulases of *C*. *gigas* and discuss their physiological and ecological significance.

## Materials and methods

### *Crassostrea gigas* collection and dissection

Adult *C*. *gigas* individuals (with shells longer than 5 cm) attached to the embankment at Niyu Fishing Harbor in Fukui Prefecture, Japan (35°42’38.7” N 135°58’16.9” E) were collected on June 11, 2021. They were then stored in local seawater before being transferred on ice. No permits were required for collecting oysters at this public site, as stated by the Fukui Prefecture Government in Japan (https://www.pref.fukui.lg.jp/). These attached individuals are wild-grown and do not belong to any interest groups.

### Total RNA and genomic DNA extraction and purification

Digestive glands, where cellulases are known to be expressed, were used to extract total RNA. Approximately 100 mg *C*. *gigas* were immediately dissected upon arrival at the laboratory, rinsed with 20 mL distilled water in a Petri dish, dried with tissue paper, surface sterilized using 70% ethanol, and homogenized with scissors. Subsequently, 1 mL TRIzol reagent (Thermo Fisher Scientific, Waltham, MA, USA) was added for total RNA extraction and purification, following the manufacturer’s protocol. The adductor muscle and mantle of *C*. *gigas* were used to extract genomic DNA, as these tissues were considered to have the fewest symbiotic organisms. They were separated, rinsed with 20 mL distilled water in a Petri dish, dried with tissue paper, surface sterilized using 70% ethanol, and immediately frozen in liquid nitrogen in a pre-frozen mortar for better homogenization. Approximately 100 mg of powdered adductor muscle or mantle was added to 1 mL TRIzol reagent, and genomic DNA was extracted following the manufacturer’s protocol.

### cDNA library

A portion of the purified total RNA from *C*. *gigas* digestive glands was used to synthesize first-strand cDNA by using the PrimeScript 1st strand cDNA Synthesis Kit (Takara Bio, Kusatsu, Japan) following the manufacturer’s protocol. The concentration of synthesized cDNA was measured by using a Nanophotometer N60 (Wakenbtech, Kyoto, Japan), dispensed to 50 μL (10 ng/μL), and stored at −70°C before use.

### Transcriptome analysis

The extracted total RNA was sequenced by an external company (Azenta, Burlington, MA, USA). Briefly, tagmentation was performed using the Nextera XT DNA Library Prep Kit (Illumina, San Diego, CA, USA) according to the manufacturer’s protocol. Next, a first-step 12-cycle PCR amplification was performed using Nextera PCR Master Mix (Illumina) and Nextera-DNB Conversion Primer (Illumina), followed by a second-step 12-cycle PCR amplification to enrich the samples and add barcode sequences. The library DNA concentration was measured using the Qubit dsDNA HS Kit (Thermo Fisher Scientific) with a Qubit 3.0 fluorometer (Thermo Fisher Scientific). Next, the library DNA quality was checked using a Sensitivity DNA Kit (Agilent Technologies, Santa Clara, CA, USA) with an Agilent 2100 Bio-Analyzer (Agilent Technologies) before circularization using the MGIEasy Circularization Kit (MGI Tech, Shenzhen, China). Finally, 20 μL (49.3 ng/μL) dsDNA was applied to a DNBSEQ-G400RS High-throughput Sequencing Set (MGI Tech) before being loaded into the DNBSEQ-G400 Sequencer (MGI Tech) and sequenced with 200 bp paired-ends.

Raw sequence data were analyzed using Atria software (v3.2.1; https://github.com/cihga39871/Atria) to trim adapters and transposon sequences. Trimmed files were analyzed using FastQC software (version 3; https://github.com/s-andrews/FastQC) to check the quality of the reads. Next, Trinity software (v2.15.0; https://github.com/trinityrnaseq/trinityrnaseq/releases) was used to assemble the sequence reads. The assembled transcriptomes were analyzed using BUSCO software (version 5.4.5) to check the quality of the assembly. Finally, functional transcript annotation was performed using the Trinotate software (v3.2.2; https://github.com/Trinotate/Trinotate/releases), particularly for similarity-based searches that referenced the Basic Local Alignment Search Tool (BLAST) and SwissProt protein sequence databases. Annotated transcripts that have similarities with cellulase (β-1,4-glucanase and β-glucosidase) were manually selected for further analysis.

### cDNA cloning of candidate cellulase open reading frames (ORFs)

All transcripts similar to cellulase were collected and their ORFs were analyzed using the Translate tool operated by the Swiss Institute of Bioinformatics (https://web.expasy.org/translate/) (Lausanne, Switzerland). Specific primers complementary to sequences covering the start and end codons of the ORF were designed according to the transcripts (Table 1). PCR amplification was performed using cDNA as the template. The amplified PCR products were separated on a 1% TAE agar gel and imaged using a UV imaging system. For one transcript that had an incomplete ORF (CgCel9D), an oligo-dT primer was designed, the amplified PCR product was purified using the NucleoSpin Gel and PCR Clean-up Kit (Takara, Kusatsu, Japan), and then sequenced using a Sanger sequencer (Applied Biosystems 3730; Thermo Fisher Scientific). With the 3′-end of the ORF verified, a new primer was designed and the complete ORF was amplified. For the PCR reaction, a 10 μL reaction mixture was used, containing 10 ng cDNA, 0.2 mM dNTPs, 1 mM MgSO_4_, 0.3 mM primers, and 0.2 U KOD plus ver.2 DNA polymerase (Toyobo, Japan). PCR products were separated and imaged as previously described.

**Table 1:** Cellulase orthologs detected and analyzed from the transcriptome of *Crassostrea gigas*.

GHF	Gene Name	Trinity ID (S4 File)	Assembled contig length (nt)	Longest ORF (nt/aa) (S4 File)	Primer (cDNA PCR)	cDNA product length (approx. bp)	Primer (genomic PCR)	Targeted length (bp)	Product length (approx. bp)	Intron existence
GHF5	*CgCel5A*	DN39864_c7_g1_i5	4,183 3′5′frame	3,183/1,061	FW: 5′-ATGGTAAAATGGCAAGTCCTG-3′ RV: 5′-TTAAGTCCGTTCGTATATAAATAG-3′	1.6 kb	FW: 5′ ATAGGAATGCACGCTAGATTG 3′ RV: same as cDNA PCR	1,060	2.5 kb	Yes
	*CgCel5B*	DN51349_c0_g1_i1	346 5′3′frame	138/46	FW: 5′-ATGGGACTGGTGTTGTTTCAC-3′ RV: 5′-CTACAACCACAACGGCTTC-3′	Amplification failed	FW/RV: same as cDNA PCR	138	Amplification failed	No
					FW: 5′-CTCCATATATCCCAGAATCCC-3′ RV: 5′-CTCAGGGTTTTTGTATACATG-3′	Nested PCR failed				
	*CgCel5C*	DN37314_c0_g1_i3	3,865 5′3′ frame	2,841/947	FW: 5′-ATGACAACCCCCGAGGGACGG-3′ RV: 5′-TTACGAGGACATCTCGCCCTC-3′	> 2 kb	FW: 5′ GCCCTACTTCTCTCCTTGGTG 3′ RV: same as cDNA PCR	690	2.2 kb	Yes
GHF9	*CgCel9A*	DN31237_c2_g1_i3	1,798 5′3′ frame	1,713/571	FW: 5′-ATGATTAGAATAGCCACCTGTC-3′ RV: 5′-TTAACTCAACATGCCACG-3′	1.7 kb	FW/RV: same as cDNA PCR	1,713	> 20 kb	Yes
	*CgCel9B*	DN34378_c0_g1_i2	1,911 5′3′ frame	1,815/605	FW: 5′-ATGTCAACAACGCACGTGG-3′ RV: 5′-CTACGTGACACAGTTCTTAATC-3′	1.8 kb	FW: 5′ GCACAGGTTGGAAAGGGTAA 3′ RV: same as cDNA PCR	1,060	2.8 kb	Yes
	*CgCel9C*	DN35380_c0_g1_i1	2,176 5′3′ frame	1,713/571	FW: 5′-ATGATGGATTCGAGCCTCG-3′ RV: 5′-TCACATTCTCAGGTGCTTC-3′	1.7 kb	FW: 5′ CTGGAGAAAATGTACGACTGTATC 3′ RV: same as cDNA PCR	1,100	3.1 kb	Yes
	*CgCel9D*	DN40142_c5_g1_i5	1,677 (fragmental)	1,659/553 (fragmental)	**For fragment ORF** FW: 5′-ATGCTCCGTTTCTGGGCGTTC-3′ RV: 5′-ACTGGGGCCAGGGTTGTTG-3′	1.6 kb	FW: 5′ CACGTGCTCGCCTATGGTCTG 3′ RV: 5′ ACTGGGGCCAGGGTTGTTG 3′	960	4 kb	Yes
			/	/	**For verifying 3′ sequence (oligodT primer)** FW: 5′-ATGCTCCGTTTCTGGGCGTTC-3′ RV: 5′-CCACGCGTCGACTAGTACTTTTTTTTTTTTTTTTT-3′	1.9 kb				
			1,921 5′3′ frame	1,074/358	**For verifying full-length cDNA** FW: 5′-ATGCTCCGTTTCTGGGCGTTC-3′ RV: 5′-TTTAAAGATATACAATTTATTTCTC-3′	1.9 kb				
	*CgCel9E*	DN22805_c0_g1_i3	1,233 5′3′ frame	705/235	FW: 5′-ATGTGTGTCAGTGCCGCCCTTC-3′ RV: 5′-CTAGCATGCAGGTGTTGGAGC-3′	0.4 kb	FW/RV: same as cDNA PCR	705	Amplification failed	No
					FW: 5′-CATTTGCTAAGGCACACCAA-3′ RV: 5′-CATGCGGCTGTTCATATGTC-3′	Nested PCR failed				
GHF45	*CgCel45A*	DN28824_c2_g1_i9	695 3′5′ frame	630/210	FW: 5′-ATGTGGTCCTTATCTGTTTTC-3′ RV: 5′-TCAATTTTCCGAGCCGTTCG-3′	0.4 kb	FW/RV: same as cDNA PCR	630	> 20 kb	Yes
	*CgCel45B*	DN28824_c2_g1_i13	393 3′5′ frame	369/123	FW: 5′-ATGACATTATACGAAAAAGGTGG-3′ RV: 5′-TCAGAATCCATTTTCCGAG-3′	Amplification failed	FW/RV: same as cDNA PCR	369	Amplification failed	No
					FW: 5′-GTTTAAAGCAGCCATGACATTATAC-3′ RV: 5′-ATAACAGAATCAGAATCCATTTTC-3′	Nested PCR failed				

### Genomic PCR

PCR of genomic DNA was first performed using the same primers as those used for cDNA cloning. Additional primers, complementary to the middle section of the ORF, were designed for PCR products containing introns that were too long for conventional PCR (Table 1). The PCR reaction used a 10 mL reaction mixture, similar to that for cDNA cloning, except that 10 ng mantle/adductor muscle genomic DNA was used as a template.

### Construction of a eukaryotic endogenous cellulase library

Endogenous cellulases in eukaryotic organisms were confirmed from the CAZy database (http://www.cazy.org/), and collated protein sequences were collected from the NCBI database (https://www.ncbi.nlm.nih.gov/) based on two rules. Firstly, only cellulase sequence data with identified full-length ORFs were collected, while fragmental sequences were ignored. Secondly, only cellulase sequence data from published references with clear endogeneity verification processes were collected. DNA/protein sequence data submitted without any information related to the risk of contamination were ignored. The collected cellulase sequences were taxonomically classified into glycoside hydrolase families (GHFs) for further analysis, as provided in the CAZy database.

### Phylogenetic analysis of *C*. *gigas* endogenous cellulases

Full-length ORFs of the endogenous cellulases of *C*. *gigas* were first translated into protein sequences and then aligned with all collected cellulase sequences in the constructed library using the MAFFT algorithm (multiple alignment using fast Fourier transform; https://mafft.cbrc.jp/alignment/software/) to confirm their closest similarity to a GHF. Next, the original (unaligned) protein sequences of each *C*. gigas cellulase were combined with the dataset of their most similar GHF and aligned using the same algorithm mentioned above. The aligned dataset of each GHF was then manually checked, and regions with long gaps were deleted. The sequences used for phylogenetic analysis are available in the supplementary data (S1–S3 File). Phylogenetic analyses were performed using IQ-TREE software (version 2.2.0; https://www.iqtree.org), applying common empirical amino acid exchange rate matrices (LG, general matrix) and common rate heterogeneity across site models (+I+G, invariable site plus discrete gamma model). Clade stability was evaluated using 100 replicates of a standard non-parametric bootstrap. Branches with less than 50 support values were collapsed. Based on the results of the phylogenetic analysis, the homology and enzymatic characteristics of the cellulases possessed by *C*. *gigas* were discussed.

## Results

### Transcriptome analysis

A total of 30,044,943 paired-end, 200 bp-long reads (12,017,977,200 bp in total) were produced after sequencing. After trimming adaptor sequences and filtering low-quality sequences using the Atria tool, 27,094,086 paired-end reads remained. The FastQC tool was used to check the quality of the reads, and the Trinity tool was thereafter used to assemble 273,631 contigs. Benchmarking Universal Single-Copy Orthologs (BUSCO) analysis (S1 Fig) showed that the assembled transcriptome mainly belonged to the Metazoa and mostly comprised completed transcripts (n = 954). Thus, the transcriptome of *C*. *gigas* was successfully completed.

### Endogenous cellulases of *C*. *gigas*

**Fig 1:**
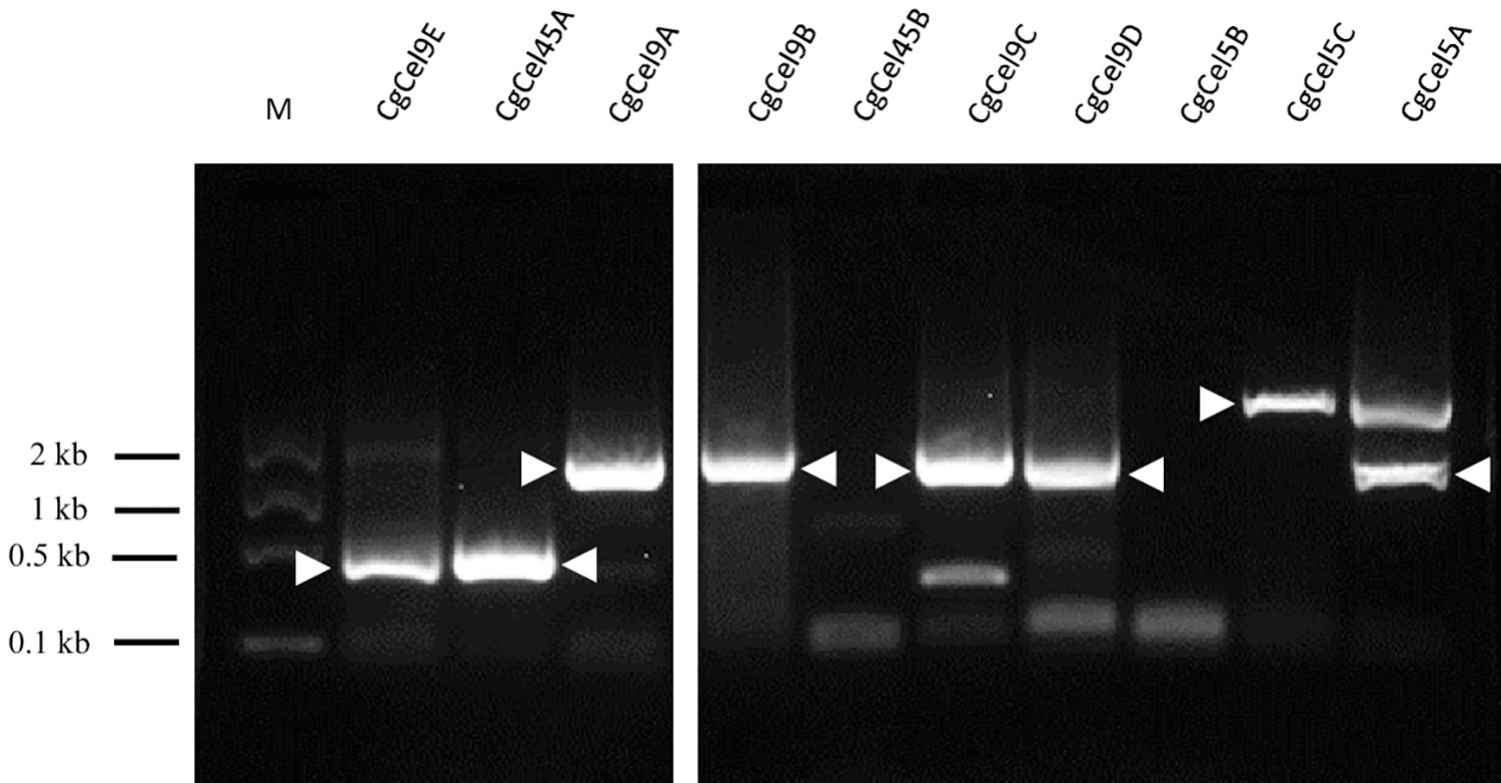
The cDNA PCR of 10 cellulase orthologs of *Crassostrea gigas* using first-strand cDNA as a template. White arrows show the major product. The primers used and the approximate size of the products are summarized in Table 1. M = Gene Ladder Fast 1 (0.1–2 kb) (NIPPON GENE, Tokyo, Japan).

As shown in Table 1, using a similarity-based search, 10 transcripts were detected as cellulase orthologs (S4 File). Among these, seven (CgCel5A, CgCel5C, CgCel9A, CgCel9B, CgCel9C, CgCel9D, and CgCel45A) were successfully amplified via PCR using digestive gland cDNA as the template, indicating successful assembly (Fig 1). For the other three orthologs that failed to be amplified during cDNA PCR, nested PCR using primers targeting the outer region of the ORFs was performed to avoid incorrect assembly and/or low target expression levels; however, all amplification attempts failed.

**Fig 2:**
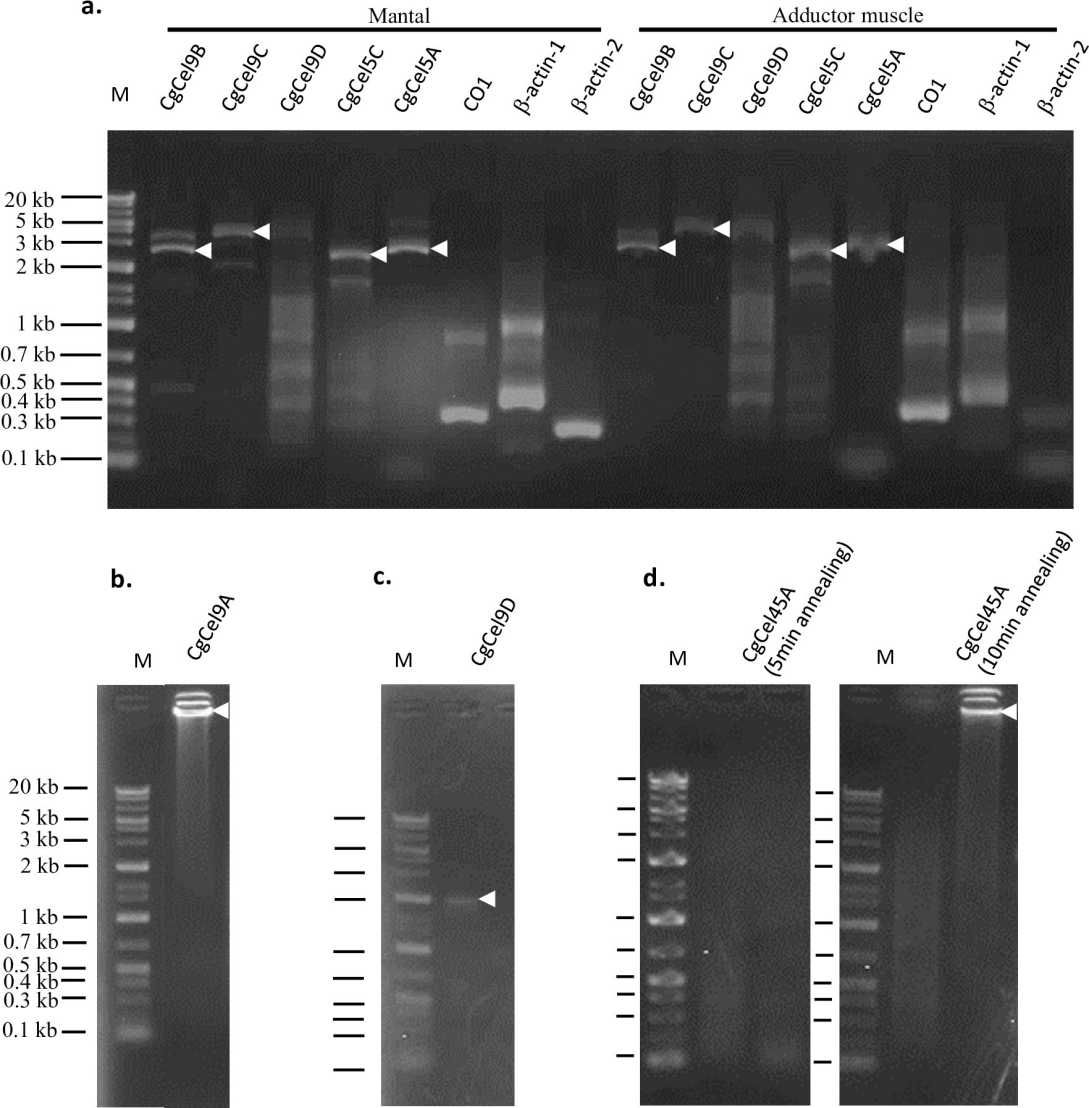
Genomic PCR of 10 cellulase orthologs using mantle and/or adductor muscle DNA of *Crassostrea gigas* as templates. White arrows show the major product. The primers used and the approximate size of the products are summarized in Table 1. **(A).** Genomic PCR of CgCel5A, Cgcel5C, CgCel9B, CgCel9C, and CgCel9D. Cytochrome oxidase subunit 1 (CO1), β-actin1, and β-actin2 are positive controls. **(B–D).** Genomic PCR of **(B)** CgCel9A, **(C)** CgCel9D, and **(D)** CgCel45A. Left, 5 min of annealing failed to produce any product. Right, 10 min of annealing successfully produced a > 20-kb product. Maker sizes are shared in the lower panel (**B**, **C**, and **D**). M = Gene Ladder Wide 1 (0.1–20 kb) (NIPPON GENE, Tokyo, Japan).

Genomic PCR was performed on all 10 orthologs using primers targeting partial or full-length ORFs. As a result, seven orthologs (CgCel5A, CgCel5C, CgCel9A, CgCel9B, CgCel9C, CgCel9D, and CgCel45A) were successfully amplified from adductor muscle and/or mantle genomic DNA templates (Fig 2). Compared with the length of the targeted cDNA sequences, a larger size of the genomic PCR products from all orthologs was verified (Table 1), indicating the existence of intron sequences. However, the remaining four orthologs failed all genomic PCR amplification attempts using primers targeting either the full-length or interparts of their ORFs, indicating that they might represent misassembled contigs.

### Phylogenetic analysis

**Fig 3:**
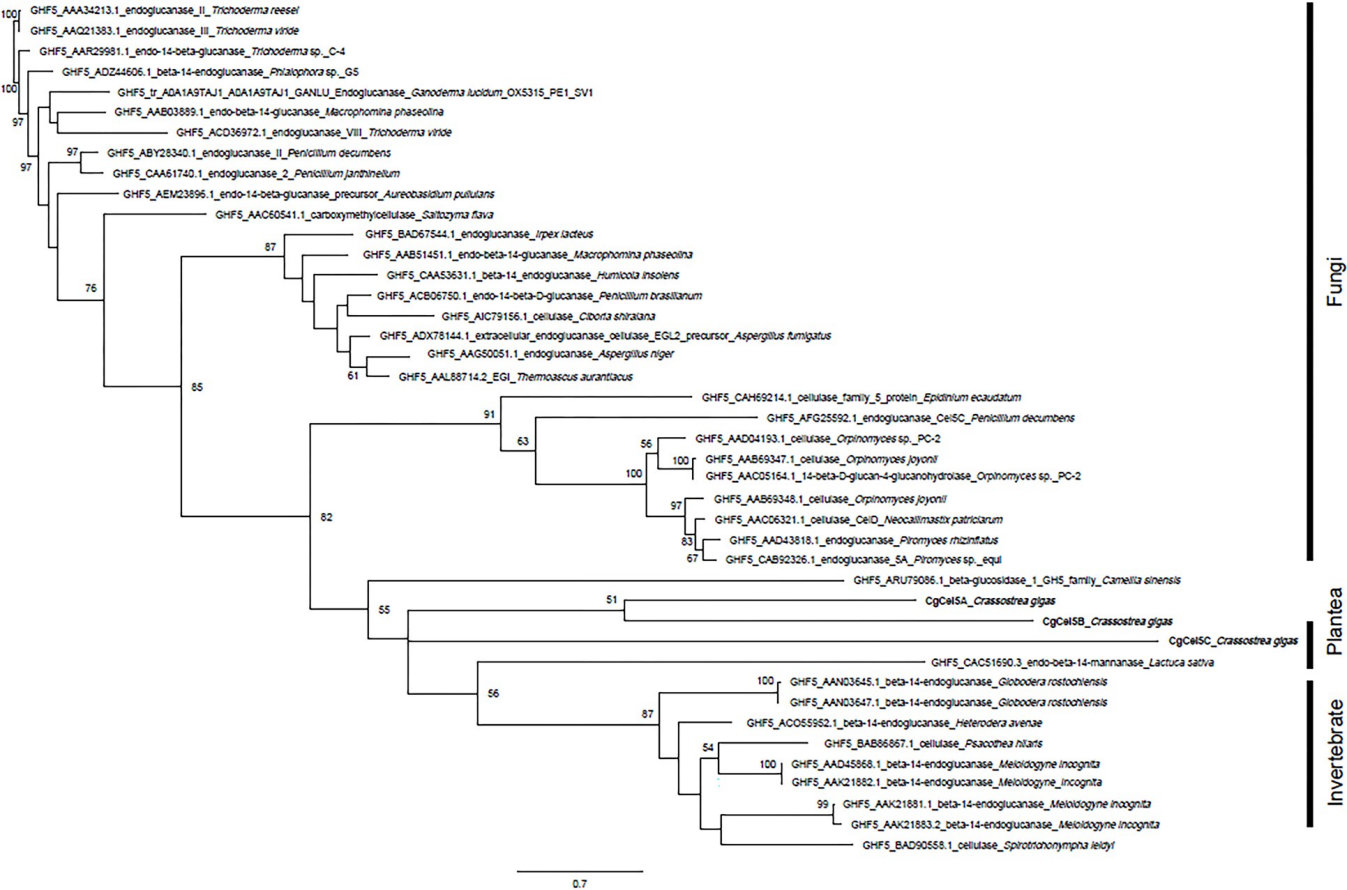
Phylogenetic position of CgCel5A, CgCel5B, and CgCel5C (in bold) in the eukaryotic endogenous cellulase gene tree of GHF5. Bootstrap values larger than 50 are shown in each branch.

**Fig 4:**
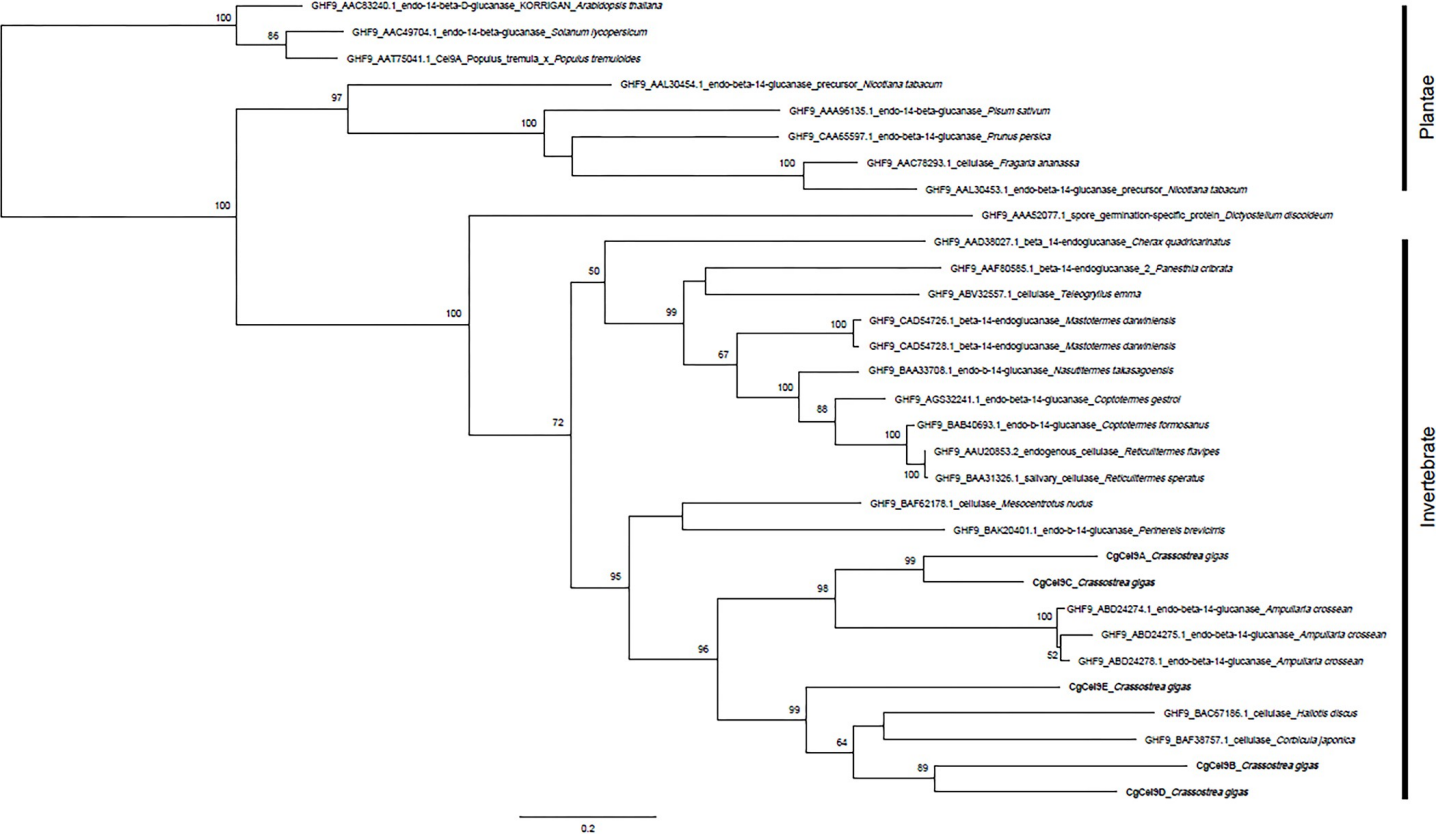
Phylogenetic position of CgCel9A, CgCel9B, CgCel9C, CgCel9D, and CgCel9E (in bold) in the eukaryotic endogenous cellulase gene tree of GHF9. Bootstrap values larger than 50 are shown in each branch.

**Fig 5:**
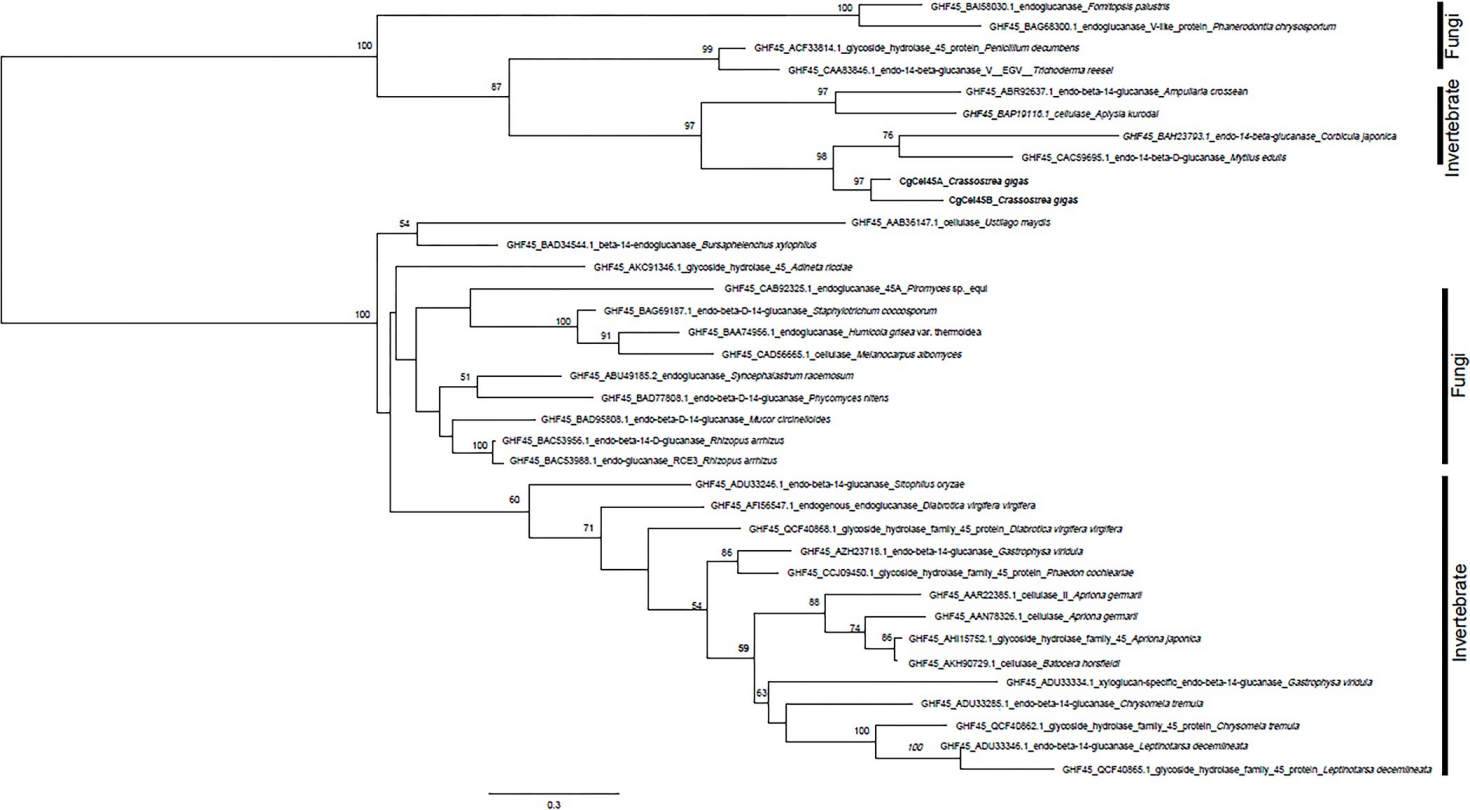
Phylogenetic position of CgCel45A and CgCel45B (in bold) in the eukaryotic endogenous cellulase gene tree of GHF45. Bootstrap values larger than 50 are shown in each branch.

Cellulase orthologs with intron sequences confirmed via genomic PCR were verified as endogenous cellulases of *C*. *gigas*. However, in the present study, we used all 10 orthologs in the phylogenetic analysis. The first round of analysis, using all data collected from the CAZy database, classified the 10 orthologs with their closest GHF based on amino acid sequence similarity (S2 Fig). The 10 cellulase orthologs belonged to three GHFs: GHF5, GHF9, and GHF45. The second round of analysis was intended to show the detailed taxonomic positions of each ortholog within its closest GHF (Figs 3–5).

For the intron-confirmed endogenous cellulases of *C*. *gigas*, CgCel5A and CgCel5C showed the highest similarity to GHF5 in terrestrial plants (Fig 3). In contrast, CgCel9A, CgCel9B, CgCel9C, and CgCel9D, which belong to GHF9, exhibited very close relationships with other aquatic invertebrates like bivalves (e.g., *C*. *japonica*) and gastropods (e.g., *Ampullaria crossean* and *Haliotis discus*) (Fig 4). CgCel45A also demonstrated the highest similarity to other bivalves (Fig 5). The remaining orthologs, for which intron sequences could not be detected via genomic PCR, were all closely related to other *C*. *gigas* orthologs in the same GHF group (i.e., CgCel5B showed the highest similarity to CgCel5A and CgCel5B [Fig 3], CgCel9E to CgCel9B and CgCel9D [Fig 4], and CgCel45B to CgCel45A [Fig 5]).

## Discussion

### Phylogenic analysis of *C*. *gigas* endogenous cellulases

Notably, the eukaryotic endogenous cellulase library constructed in this study omitted fragmental sequences to increase the reliability of the results. In addition, some invertebrates, such as termites, have eukaryotic symbiosis and possess cellulases [13]. Thus, it is critical to check the method by which each sequence is obtained and to evaluate the likelihood of "contamination" when discussing the origin of a cellulase. We only collected sequences that were extracted from published references, where the method of sample preparation or endogeneity verification is clearly stated. Finally, although this step was not performed during the library construction process, we also gathered data on whether each cellulase sequence was confirmed to possess enzymatic activity via indisputable methods like protein purification and recombinant expression. These data were taken into account when discussing the putative activity of endogenous cellulases in *C*. *gigas*.

Among the 10 cellulase orthologs found in *C*. *gigas*, CgCel5A, CgCel5B, and CgCel5C were categorized as GHF5 (Fig 3). As shown in Table 1, the cDNA PCR of CgCel5A amplified a product with a size of 1.6 kb, which was smaller than its expected ORF (3,183 bp). However, genomic PCR confirmed the presence of introns in CjCel5A, indicating that it was endogenous to *C*. *gigas*. Although we attempted to target the 138-bp ORF of CgCel5B using cDNA as a template, no PCR product could be amplified. Subsequently, we employed primers to target the outer region of the ORF and conducted nested PCR, yet we still failed to amplify a PCR product. Moreover, despite both sets of primers being used for genomic PCR, no product could be amplified. Considering the short length of the ORF, it is possible that CgCel5B represents a misassembled or incomplete (fragmental) contig. On the other hand, for CgCel5C, a 2.8 kb product was obtained through cDNA PCR, matching the target length of its ORF. Additionally, the existence of an intron was confirmed through genomic PCR. In the phylogenetic tree of GHF5 (Fig 3), *C*. *gigas* is included in a clade consisting of invertebrates and higher plants. This clade is clearly separated from fungi (bootstrap support = 82). Within this clade, orthologs of *C*. *gigas*, several invertebrates including nematodes and insects, and terrestrial plants are included. *Crassostrea gigas* should show homology to invertebrates since they are evolutionarily closer compared to plants, but the bootstrap support between them is low. According to Chang and Lai [14], the evolutionary origins of GHF5 are unclear. Invertebrates like *C*. *gigas* and nematodes may have acquired cellulase genes through either vertical inheritance from common ancestors or intermittent horizontal acquisitions from bacteria or fungi (possibly also intestinal microbiota). However, these organisms have shown low similarity in recent times. Regarding the biochemical activity of CgCel5A and CgCel5B, it is important to note that their orthologs in terrestrial plants, such as *Camellia sinensis*, have been found to possess β-glucosidase activity [15]. However, it is challenging to assume that CgCel5A and CgCel5B share the same enzymatic activity solely based on their amino acid sequences (S3 Fig). To definitively confirm their true enzymatic activity, the use of CgCel5A and CgCel5B recombinants would be the most efficient approach.

**Fig 6:**
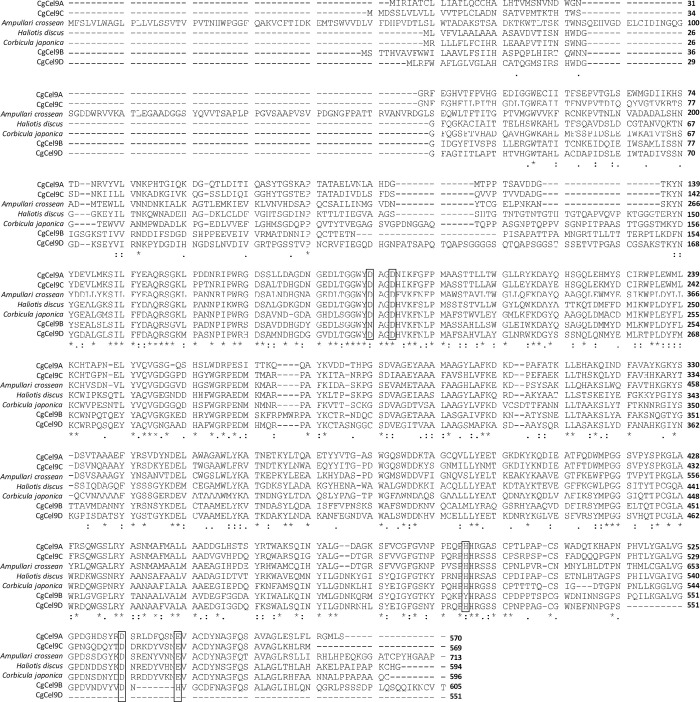
Amino acid sequence alignment of CgCel9A, CgCel9B, CgCel9C, CgCel9D, and three other GHF9 orthologs from *Ampullaria crossean*, *Haliotis discus*, and *Corbicula japonica*. Dots indicate multiple identical residues, while asterisks represent fully identical residues among each ortholog. Open squares indicate catalytically important residues, including aspartic acid, as determined by previous research [10].

Five orthologs (CgCel9A, CgCel9B, CgCel9C, CgCel9D, and CgCel9E) were categorized as GHF9, with the highest counts of endogenous cellulases in aquatic invertebrates (Fig 4). CgCel9A, CgCel9B, and CgCel9C amplified PCR products of 1.7, 1.8, and 1.7 kb in size, respectively. The sizes of these products were identical to the expected ORF lengths. In addition, the introns confirmed via genomic PCR showed endogeneity. For CgCel9D, we successfully amplified a PCR product of 1.6 kb in size, which matches the size of the assembled contig. However, as the ORF sequence inside this contig is fragmental (no stop codon), a poly-dT primer was used to verify the 3′-end sequence of the ORF. A 1.9 kb product was successfully amplified after targeting the full-length ORF. After using primers targeting the inner region of the ORF, genomic PCR amplified a 4-kb product, indicating that it is an endogenous cellulase of *C*. *gigas*. Phylogenetic analysis showed that CgCel9A and CgCel9C were very closely related to each other and had the highest similarity to an endo-β-1,4-glucanase preserved in *A*. *crossean*, a freshwater gastropod whose cellulase (ACEG65) was purified via high-performance liquid chromatography and verified to have carboxymethyl cellulose (CMC) hydrolytic activity. In contrast, CgCel9B and CgCel9D showed the highest similarity to the brackish bivalve (*C*. *japonica*) [10] and abalone (*H*. *discus*) [16]. The cellulase of the abalone was found to have lignocellulose hydrolytic activity. Amino acid alignment, which includes these orthologs, indicates that they have common catalytically important residues (Fig 6) [10]. These results showed that *C*. *gigas* has multiple GHF9 cellulases encoded in its genome, which might function similarly to that of endo-β-1,4-glucanases. It might be considered redundant to have similar cellulases encoded in the genome. However, based on our current knowledge, the same cellulase could exhibit different enzymatic activities. For example, it may have distinct functions associated with the length of the lignocellulose chain [17], and times, with the level of lignocellulose crystallites [18]. Multiple GHF9 cellulases might act differently on different sources of lignocellulose to optimize decomposition efficiency. For CgCel9E, when targeting its 1,233-bp ORF, a 0.4 kb PCR product was amplified. We then used primers targeting the outer region of the ORF and performed nested PCR, but amplification failed. In addition, both sets of primers were used for genomic PCR, but no product could be amplified. These results indicate that CgCel9E might represent a misassembled contig. Regardless, we used CgCel9E in the phylogenetic analysis, and the results showed that it was closely related to CgCel9B and CgCel9D.

**Fig 7:**
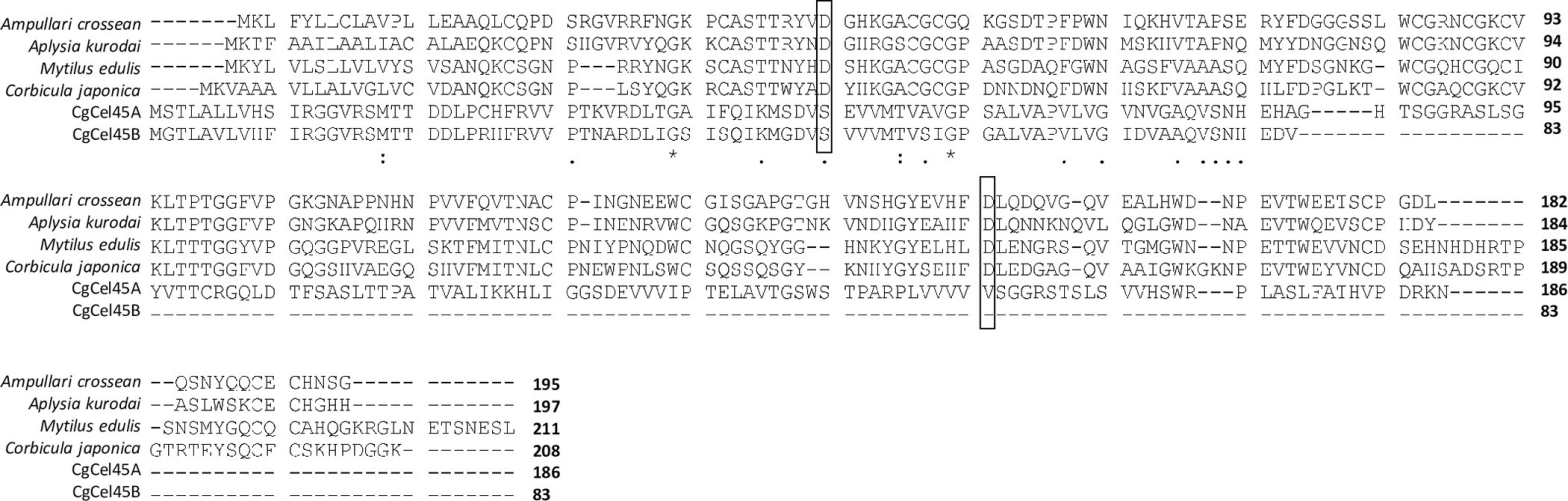
Amino acid sequence alignment of CgCel45A, CgCel45B, and four other GHF45 orthologs from *Ampullaria crossean*, *Aplysia kurodai*, *Mytilus edulis*, and *Corbicula japonica*. Dots indicate multiple identical residues, and asterisks represent fully identical residues between each ortholog. Open squares indicate the aspartic acid residue of the active center based on previous research [11].

The two orthologs, CgCel45A and CgCel45B, were categorized as GHF45. CgCel45A yielded a slightly shorter product (0.4 kb) than expected. For genomic PCR, the first amplification attempt using the same reaction conditions as those used for the other orthologs failed, but by extending the annealing time from 5 to 10 min, the second attempt successfully amplified a large product (> 20 kb) (Fig 2). However, for CgCel45B, both cDNA and genomic PCR failed to yield a product. This failure might be attributed to misassembly because the assembled sequence of CgCel45B was markedly short (only 369 bp in length). According to the phylogenetic analysis, CgCel45A had the highest similarity with the two bivalves, *Mytilus edulis* and *C*. *japonica*, indicating that this clade comprises a common endogenous cellulase possessed by aquatic invertebrates. Amino acid alignment showed that CgCel45A and CgCel45B share common residues in the active center of the cellulase with several invertebrates (Fig 7) [11]. The study of the *C*. *japonica* GHF45 cellulase (CjCEL45) revealed its ability to decompose CMC but did not verify whether it is an endo-β-1,4-glucanase, exo-β-1,4-glucanase, or β-glucosidase [11]. Unfortunately, the enzymatic activities of other cellulases of aquatic invertebrates in this clade have not yet been studied.

### Physiological and ecological significance of *C*. *gigas* endogenous cellulases

*C*. *gigas* and other oysters have empirically been found to have higher productivity when terrestrial organic matter is supplied upstream. They are able to survive in mangrove areas, even during the rainy season when the water has very low light transparency (almost 0 m) due to the large amount of insoluble particles in the water. Oysters are known to feed on phytoplankton that require light for photosynthesis, such as diatoms. However, the presence of such low numbers of primary producers did not decrease the productivity of oysters, leading some ecologists to explore the possibility that oysters could forage on particles originating from mangroves [19]. Our previous study revealed that small crustaceans (e.g., Mysidacea and Copepoda), which are heterotrophic predators that mainly feed on phytoplankton, have high levels of cellulase activity, allowing them to assimilate terrestrial lignocellulose [20]. Unlike small crustaceans, oysters, which are aquaculture species with high economic value, have been large-scale (next-generation) sequenced previously. However, few studies have focused on providing a comprehensive understanding of their endogenous cellulases, and the ecological significance of this has never been discussed. Based on the results of the present study, *C*. *gigas* has at least seven endogenous cellulases covering three GHFs. Putatively, these cellulases consist of both endo-β-1,4-glucanase and β-glucosidase, based on the database constructed in the present study. Using an online database, exo-β-1,4-glucanase was also detected. If all endogenous cellulases discovered have the expected activity, *C*. *gigas* should be capable of self-decomposing lignocellulose (e.g., broken leaves) into glucose. This capability might be the key to thriving downstream of forests or surviving in areas with low primary producers owing to low light penetration.

Studies on aquatic invertebrate cellulases are increasing, but few efforts have been made to reveal all endogenous cellulases possessed by one species. This information is essential for understanding the physiological and ecological advantages of certain species. Taking the present study as an example, possessing β-glucosidase indicates that *C*. *gigas* is more likely to use terrestrial lignocellulose as a direct food source rather than using only endo-β-1,4-glucanase and exo-β-1,4-glucanase to digest microalgae with cell walls made of cellulose. The abundance of diatoms and photosynthetic dinoflagellates is believed to reduce the need for cellulase because these microalgae are more nutritious. On the other hand, an increase in cellulose is thought to increase the expression of *C*. *gigas*’s natural cellulase, especially when these microalgae are insufficient. Other environmental factors such as pH, water temperature, and salinity can also affect the efficiency of cellulase, potentially influencing *C*. *gigas*’s cellulases positively or negatively. However, there have been no prior studies focused on the regulation of *C*. *gigas*’s or other oysters’ natural cellulase, which requires further investigation.

Additionally, carbohydrate-binding modules (CBMs) were detected in the transcriptome. Although CBMs do not have direct hydrolase activity on lignocellulose, it is necessary to mention their possible role in bivalves. CBMs have been well studied in bacteria and fungi, but there is limited knowledge about them in invertebrates [21, 22]. Our recent research focused on a Family 2 CBM of *C*. *japonica* used recombinant expression to verify its function of having a high affinity to crystalline cellulose [23]. Usually, CBMs are considered to increase cellulose decomposition efficiency. The CBM linked to the cellulase of *C*. *japonica* plays a different but important role in the decomposition of lignocellulose because it uses secreted cellulase to decompose cellulose *ex vivo* (in the sediment), thus requiring CBMs to keep the secreted cellulase anchored on the leaves. It is difficult to estimate whether *C*. *gigas* uses the same method as that of *C*. *japonica*, which inhabits the sediment of wetlands where immobilized lignocellulose (i.e., leaves) can easily be found, whereas *C*. *gigas* lives close to seawater where lignocellulose can easily be washed away. Interestingly, based on our experience in culturing bivalves (unpublished), they sometimes secrete slime-like substances, possibly extracellular polysaccharides, to capture particulate organic matter in water, including leaf fragments, where CBMs might be useful. However, the functions and distribution of CBMs in bivalves remain poorly understood and require further study to verify this assumption.

### Approach to reveal the ecological significance of aquatic invertebrates in the carbon cycle

Our previous study, which utilized zymographic assays, revealed the presence of cellulase activities not only in oysters but also in almost all aquatic invertebrate phyla, indicating the presence of endogenous cellulases [7, 24]. A recent study, based on genome and transcriptome analyses, further supported this assumption [14]. However, additional research is required to fully understand the role of the carbon cycle in aquatic invertebrates. Since it is possible for one invertebrate species to possess multiple endogenous cellulases, comprehensive studies like the present one, which thoroughly investigate the endogenous cellulases of the target invertebrates, will be necessary. In this process, it is important to carefully exclude any contamination from symbiotic organisms. Next, using phylogenetic analysis and referencing previous studies, the enzymatic activity of the newly identified cellulase orthologs was predicted. However, since studies on invertebrate cellulases are still limited and it can sometimes be difficult to predict the enzymatic activities of certain hydrolases based solely on nucleoside/amino acid sequences, methods like protein purification and recombinant expression are still necessary to confirm the activity of a detected cellulase. Following this, quantification analysis of activity-confirmed endogenous cellulases expressed in the field should be conducted in order to evaluate the amount of lignocellulose used by invertebrates, such as *C*. *gigas*, and assess its ecological significance.

## Conclusion

This study successfully identified seven endogenous cellulases. First, transcriptome data were mined to target cellulase orthologs. Genomic analysis was then conducted to confirm their existence, including the presence of introns. Three cellulase orthologs that could not be amplified by PCR using either cDNA or genomic DNA as templates were found to be a result of misassembling of the next-generation sequencing data of the total RNA or symbiotic organism contamination during the RNA extraction step. For future studies aiming to identify endogenous cellulase or other glycoside hydrolases related to decomposing terrestrial plant organic matter, it is recommended to follow our method to avoid erroneous determination of endogeneity. The identified cellulase sequences in this study could also be utilized to identify endogenous cellulase in other oyster species. Additionally, this opens up the possibility for quantitative analysis of the expression level of *C*. gigas’s cellulases. By examining the ability of oysters to decompose lignocellulose, it is now possible to investigate the differences in thriving or non-thriving oysters across different environments.

### Accession numbers

All data, including figures and tables, supporting information, and related raw data are deposited in a public repository (Dryad, https://datadryad.org/) and are fully accessible (https://doi.org/10.5061/dryad.d2547d88j).

## Supporting information

S1 File Fasta file of amino acid sequences of GHF5 (including data from the present study) that were modified and used in the phylogenetic analysis. (FAS)

S2 File Fasta file of amino acid sequences of GHF9 (including data from the present study) that were modified and used in the phylogenetic analysis. (FASTA)

S3 File Fasta file of amino acid sequences of GHF45 (including data from the present study) that were modified and used in the phylogenetic analysis. (FAS)

S4 File Assembled cDNA and longest translated amino acid sequences of the 10 cellulase orthologs. (XLSX)

S1 Fig BUSCO analysis of the assembled contigs. (PDF)

S2 Fig Phylogenetic position of all 10 cellulase orthologs in the merged gene tree of GHF1, 3, 5, 6, 7, 9, 12, and 45. (PDF)

S3 Fig Amino acid sequence alignment of CgCel5A, CgCel5B, and three other GHF5 orthologs from *Camellia sinensis*, *Lactuca sativa*, and *Globodera rostochiensis*. Dots indicate multiple identical residues shared by each ortholog.
(PDF)
