# Supplementary figures and images for: Comprehensive cDNA cloning and putative feature analysis of endogenous cellulases possessed by the Pacific oyster, Crassostrea gigas
Source: PLoS One. 2025 Feb 7;20(2):e0313246. doi: 10.1371/journal.pone.0313246 (PMC11805347; doi:10.1371/journal.pone.0313246)

S1 Fig.

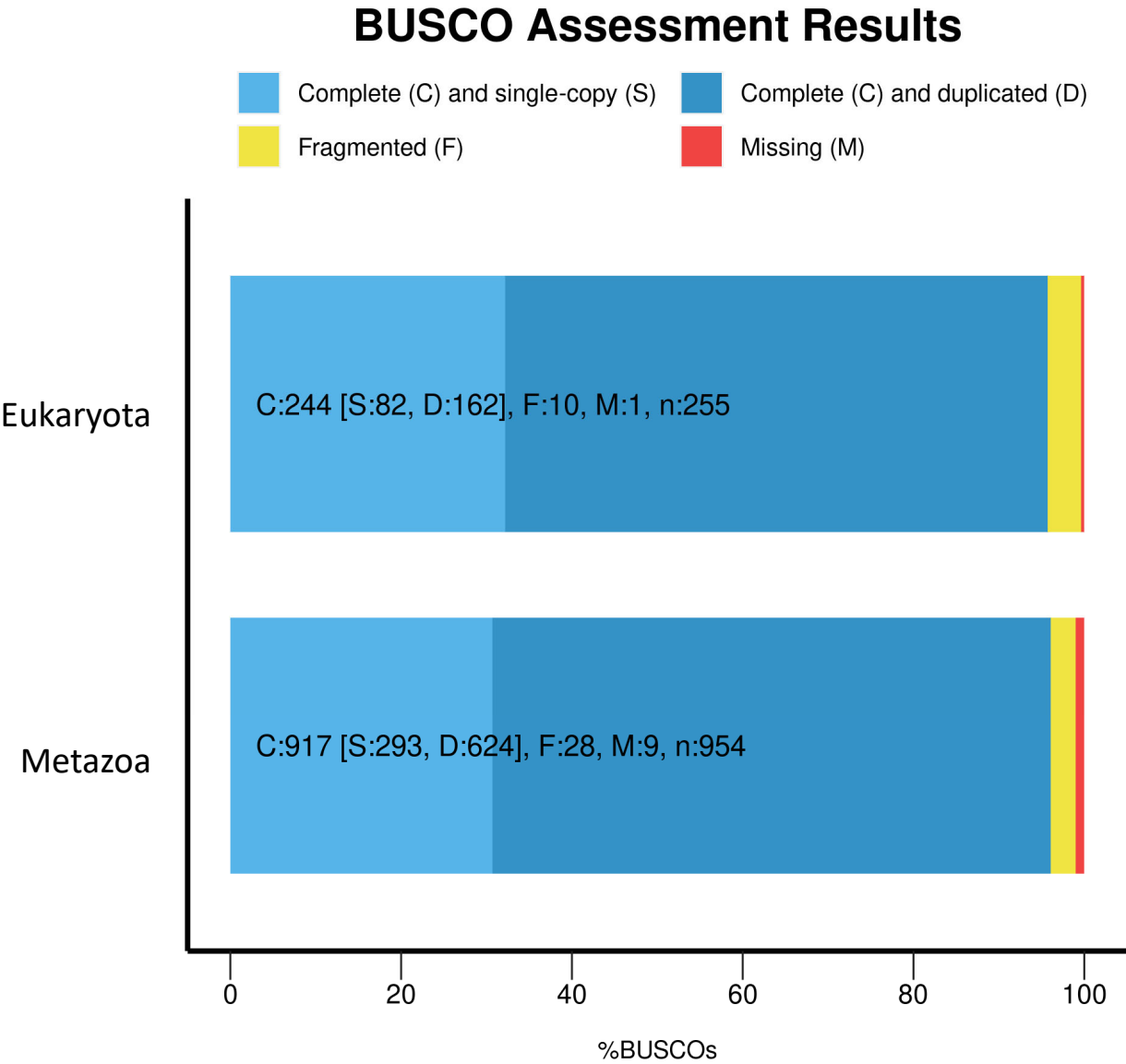

Supplement: S1 Fig — (PDF) [file pone.0313246.s005.pdf]
