# Supplementary material for: Comprehensive cDNA cloning and putative feature analysis of endogenous cellulases possessed by the Pacific oyster, Crassostrea gigas
Source: PLoS One. 2025 Feb 7;20(2):e0313246. doi: 10.1371/journal.pone.0313246 (PMC11805347; doi:10.1371/journal.pone.0313246)
Supplement: S3 Fig — Dots indicate multiple identical residues shared by each ortholog. (PDF) [file pone.0313246.s007.pdf]

S3 Fig.

```
      Camellia sinensis ---------- ---------- ---------- ---------- ---------- ---------- ---------- ---------- ---------- ---------- 0
Globodera rostochiensis ---------- ---------- ---------- ---------- ---------- ---------- ---------- ---------- ---------- ---------- 0
         Lactuca sativa ---------- ---------- ---------- ---------- ---------- ---------- ---------- ---------- ---------- ---------- 0
                CgCel5A MVKWQVLWYL IGLHCSLIHA ALPPDCVPLE EHPLDTADPG SIFGETSEVG SKASPHRTGN LRQRGAIPTH HFAANEIKSA EPVPMRLFPY EAIMTSSGIV 100
                CgCel5B ---------- ---------- MTTPEGRRLK EDRTRAKNWK RWGPYLSERQ WGTVREDYSD DGNCWNYFPH DHARSRAYRW GEDGLLGICD REGRLCMGVA 80

      Camellia sinensis ---------- ---------- ---------- ---------- ---------- ---------- ---------- ---------- ---------- ---------- 0
Globodera rostochiensis ---------- ---------- ---------- ---------- ---------- ---------- ---------- ---------- ---------- ---------- 0
         Lactuca sativa ---------- ---------- ---------- ---------- ---------- ---------- ---------- ---------- ---------- ---------- 0
                CgCel5A YDAYTMDRWT DPERTDLMMQ QSAWKSGEPW NKLTDMGTNM RQVVSGPDGN PGGVFSGKSG VQVGISGSSD PVMTDFGDLY ANFEYHGNGG KMEVPLVRGS 200
                CgCel5B LWNTKDPILK ERLFGLTGHQ GNHGEDVKEL YYYLDSTPTH SYMKALYKYP QNEYPYSQLE NENRIRGFMM PEYEILDTGV IDQGYFDVMA EYSKHTPNDV 180

      Camellia sinensis -----MATSS TRQLLGICFF LCYMLSLSD- -----GRVTP NFKIRAVNLG GWLVTEGWIK PSLFDGIPNK DFLDGTGLQF KSVTVGKFLC AEQGGGTI-- 87
Globodera rostochiensis ------MCAL IYAVLPLLLV LIADHSKIG- -----AVTAP PYGPLAVNG- -KFLVQKSTK KTVKLHGLSL DWSQWQPRFW VAETVNRIKC GCNSN----- 81
         Lactuca sativa -----MPGKG NAVLKGDCKG KCHLSTIND- -----SFVET RTHHFMVNGK PLYFNGFNAY WLMCMASDPS TRVKVTDAFQ ESSKIGMN-- ---------- 77
                CgCel5A ALLTHLFTNA NPVVKPFCLA DISGHHVNFN CPLERTILDA GSGHASATCS GGVLHLRLQN TKRIRDVTKI QYAARPKTRW AAGDHPMSSC DVNKCRLTDG 300
                CgCel5B LAKYTVTNRG KERATVHVLP TLWFRNVWSW GEDAYCYVSP KPHLSQVSGC KVKCDHPTLG EDQSHHIDPT VDQATPPPAF IGSFYWEVDK DQSNKEPELL 280
                                                                  .       ..                                     .

      Camellia sinensis ---------- ---------- ---------- ---------- ---------- ---------- ---------- ---------- ---------- ---------- 87
Globodera rostochiensis ---------- ---------- ---------- ---------- ---------- ---------- ---------- ---------- ---------- ---------- 81
         Lactuca sativa ---------- ---------- ---------- ---------- ---------- ---------- ---------- ---------- ---------- ---------- 77
                CgCel5A GKTLEVDVPN AHGTMAFAFN YIGHYVTPWD WINHPEEVTC HGRRSEKRAP NDITLHASCD SNLNLEIGVY VGHHHIYGVD KIQYAVEPEN TWGNPPPMHT 400
                CgCel5B FTENETN--- ---------- ---------- ---------- ---------- ---------- ---------- --HKRLFNVD GQHYSKDAFH NYVIKGDKSA 315

      Camellia sinensis ---------- ---------- ---------- ---------- ---------- ---------I VANRTSASGW ETFKLWRINE TTFQFRVFNK QFMGLNYATG 128
Globodera rostochiensis ---------- ---------- ---------- ---------- ---------- ---------- -VVRAAMGTQ RSFGGYVANP ----AAEYAK MKTIIEAALD 116
         Lactuca sativa ---------- ---------- ---------- ---------- ---------- ---------- -LVRTWAFSD GGNKPLQTSP GFYNEDMFKG LDFVISEAKK 116
                CgCel5A CNPTVCTRVG NTIYVRIHAP AQHLKLAVNV IGYVTLPAFW ISHPYKMVCG GPDIGSSVSG GHPVTPAPTI HTFPTSQTQP THTQPPQTQP LPSIRTTASS 500
                CgCel5B VNPSHCGTKC AAHYVLTLDP GQEFTIKVRL YFQLEAPKTE VFGPDFDEIF QDRQKEADLF YDEKIPMRDQ KQRQVSRQAY AGLLWSKQFY YYVIEEWLKG 415

      Camellia sinensis ----NGIDVV AIANTSSETF -----QIVRN SDDLSRVRIK APNGFFLQVK TEELVTADSK GDGGWGDSDP SVFIMTISGR LQGEFQVTNG YGPLKAPQVM 219
Globodera rostochiensis ----QGIYVI VDWHTG---- ---------- -DDLATDEIN SAKEFFTKIA QTYGKHP-HI IYEIWN---- ---------- --------EP LKQTTTWEAV 174
         Lactuca sativa ----YGIHLI LSLVNNWDDF G-GKKQYVQW ARDHGGQYLN SDDDFFTNVV VKGYYKNHLK TILTRRNSIT GVDYKDDSTI FSWELMN-EP RCQSDLSGKF 210
                CgCel5A RPITQGSKFI LELNEPGDEL PNQTRKFLLY FQSIVVPHIN DADNTITFTP QNGGTYSGFM QLAYLGAGLR GDHSNDTVLD KHLGVYSYKP KASYCVQNNR 600
                CgCel5B DSTQPPPPAS RLKGRNFEWL HLFNMDIISM PDKWEYPWYA SWDLAFHMIP MADIDVQFAK DQLLLFLREW YLHPNGQLPA YEFSFQDVNP PVHAYAVLRV 515
                                                           .          .  :                                              :

      Camellia sinensis REHWSTFIVE EDFKFISNNG INAVRIPVGW WIASDSKPP- ---------- ---------- ---------- ---------- ---------- ---------- 258
Globodera rostochiensis IKPYSKTMVE LIRKYDKNN- VIIVGTPN-W DQDVDIVAK- ---------- ---------- ---------- ---------- ---------- ---------- 211
         Lactuca sativa LQEWIVEMAA EIKSIDKNHL LEIGLEGFYG ESMPEKKQN- ---------- ---------- ---------- ---------- ---------- ---------- 249
                CgCel5A AYASFDWNPN NQYPYQSSGS LLMITMPHHA YILKDQFAAN LKSTVYTFKG YEGPSWLLDM EAPRAAMEPD PVGVQAIKAN SQHLQDILQA IERDASQANL 700
                CgCel5B YKASGPKNKR DLAFLARCFH KLILNFTWWI NRKDLEGRNI FTGGFLGLDN IG-------- ---------- ---------- ---------- ---------- 567

      Camellia sinensis ---------- --------HP YVGGSLQALD NAFLWSRKYD IKVIIDLHAA PGSQNGFEHS SPRDGFQEWG LSDQNIQQTV DVIDFLTARY ANNPSLYAVE 340
Globodera rostochiensis ---------- --------SP LTG------- ---------Y SNIAYTLHFY AGQHN----- -------EWL RTRTKTAYNL GLPMFVTEYG IYS------- 258
         Lactuca sativa ---------- --------NP GYEVGTDFIT NNGVNNVDFA TIHMYPDQWV PGASDEAR-- --AKFVEKWI NAHIEDCDSI LRKPLLIAEF GKSS------ 321
                CgCel5A EAYCSFSDSY GVGKGIGMHA RLASISRAFG TSHYQALDGK IRHCLEKWLR IDDTLPERNR FHYDTVWGGL FLRGEDGEAH FYTDFGFPFY NDHHFHLGYF 800
                CgCel5B ---------- ----IFDRSK PLPTGGYLVQ ADATAWMAFF CSIMLEISLT LSRRDCIYED MASKFFEHFV AIADAINQKD GIGLWNEEDG FFYDHIRVNG 653
                                                                                .

      Camellia sinensis LINEPLAPGA SLDSIIKYYK AGYETVRKHS KTAYVVMSNR LGQVDPKELF PLASG----- ---------- ----LERTVI DVHYYNLFSD VFNDMTVQQN 421
Globodera rostochiensis ---EPKNEAN NLKELALWYK LLDSLSMSYA AWQVTDINEQ HAMFTPG--- ---------- ---------- ---------- ---------- --------VT 304
         Lactuca sativa -WSSGYTVEA RDEYFGGIFN TAYESARNRG SCSGTTFWQV MAEGMDNWG- ---------- ---------- ---------- ---------- ------DGYQ 373
                CgCel5A LYALAYYVRH DTAWAQQHRQ RIYALARDVG NPSYKDKFFP VVRHKDIYMG ISWASGVVPG ERQEESSSES LNCYHGLAAL GDALNDPILR GTGQVMLALE 900
                CgCel5B KKTMPLKILS MVGLVPLFSC MVLKQDVLRR HPGFYKRTRW FLNNRKDLSH HISFMCD--- -----NEDTE EPALLLSLVK KEQLQRILAH VLDENKFLSP 745

      Camellia sinensis INFINTNRTA DLNLVTTSNG PLTFVGEWVA EWQVSGATKE DYQKFAEVQL QVYGRATFGW AYWTLKNVQN HWSLQWMIQN GYIKL----- ---------- 506
Globodera rostochiensis INNICN---- ---------- ---------- ---------- ------PAYL TTYG----KY IYNKLKSQNN GVSCRG---- ---------- ---------- 336
         Lactuca sativa VVLD------ ---------- ---------- ---------- ------QNPS TAAIIAKQSQ RISSLNSSTN LEFSPVAVD- ---------- ---------- 410
                CgCel5A IASVREYYHV RDHNFNQFPP ILQKFGAVGQ IAEDSFYVYT LDWGCDPNVF PMRHGCLVGI QIIPITAVSR YWMDKDWAKH ILQSCDWAIN PS-------- 992
                CgCel5B YGIRSLSKHH RKEPFILEAG GNAYGVKYEP GESESNMFGG NSNWRGPIWF PMNYLLIENL RRYDYFFGDS LKVECPTGSG KMMRLTDVAH ELSLRLSRLF 845

      Camellia sinensis ---------- ---------- ---------- ---------- ---------- ---------- ---------- ---------- ---------- ---------- 506
Globodera rostochiensis ---------- ---------- ---------- ---------- ---------- ---------- ---------- ---------- ---------- ---------- 336
         Lactuca sativa ---------- ---------- ---------- ---------- ---------- ---------- ---------- ---------- ---------- ---------- 410
                CgCel5A ---------- HATDYSLANP SDLRDLVTGW KAFCYAGIAP YDESHKIAAA NYLRDKYPRD LVSGTGAAST LLFIYERT-- ---------- ---------- 1060
                CgCel5B YPSPEGFRPC HGDEEKYAKD PNFKDYVLFY EYFHGDSGRG CGASHQTGWT ALVANLQKYI AIREKWISSV HMAQLRKISN TGIPLDIDIM MDFSSEGEMS 945

      Camellia sinensis - 506
Globodera rostochiensis - 336
         Lactuca sativa - 410
                CgCel5A - 1060
                CgCel5B S 946
```
