# Supplementary material for: Comprehensive cDNA cloning and putative feature analysis of endogenous cellulases possessed by the Pacific oyster, Crassostrea gigas
Source: PLoS One. 2025 Feb 7;20(2):e0313246. doi: 10.1371/journal.pone.0313246 (PMC11805347; doi:10.1371/journal.pone.0313246)
Supplement: S2 Fig — (PDF) [file pone.0313246.s006.pdf]

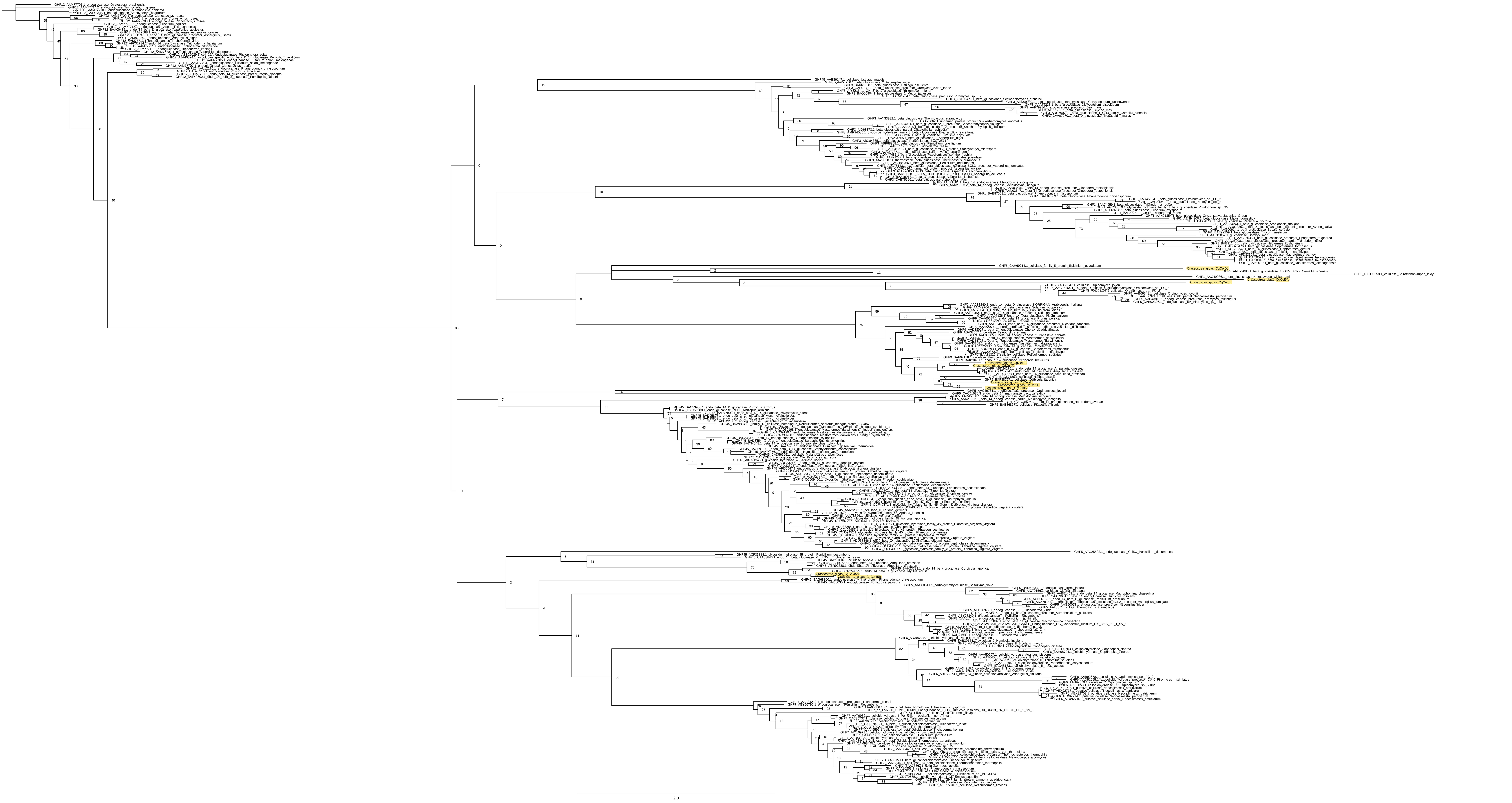
2.0
